# Variables associated with children's physical activity levels during recess: the A-CLASS project

**DOI:** 10.1186/1479-5868-7-74

**Published:** 2010-10-12

**Authors:** Nicola D Ridgers, Stuart J Fairclough, Gareth Stratton

**Affiliations:** 1 Centre for Physical Activity and Nutrition Research, Deakin University, 221 Burwood Highway, Burwood, Victoria 3125, Australia; 2 Faculty of Education, Community and Leisure, Liverpool John Moores University, I.M. Marsh Campus, Barkhill Road, Liverpool, L17 6BD, UK; 3 Research Institute for Sport and Exercise Sciences, Liverpool John Moores University, Tom Reilly Building, Byrom Street, Liverpool, L3 3AF, UK; 4 Research in to Exercise, Activity and Children's Health (REACH) Group, Liverpool John Moores University, UK

## Abstract

**Background:**

School recess provides a daily opportunity for children to engage in physically active behaviours. However, few studies have investigated what factors may influence children's physical activity levels in this context. Such information may be important in the development and implementation of recess interventions. The aim of this study was to investigate the association between a range of recess variables and children's sedentary, moderate and vigorous physical activity in this context.

**Methods:**

One hundred and twenty-eight children (39% boys) aged 9-10 years old from 8 elementary schools had their physical activity levels observed during school recess using the System for Observing Children's Activity and Relationships during Play (SOCARP). Playground variables data were also collected at this time. Multilevel prediction models identified variables that were significantly associated with children's sedentary, moderate and vigorous physical activity during recess.

**Results:**

Girls engaged in 13.8% more sedentary activity and 8.2% less vigorous activity than boys during recess. Children with no equipment provision during recess engaged in more sedentary activity and less moderate activity than children provided with equipment. In addition, as play space per child increased, sedentary activity decreased and vigorous activity increased. Temperature was a significant negatively associated with vigorous activity.

**Conclusions:**

Modifiable and unmodifiable factors were associated with children's sedentary, moderate and vigorous physical activity during recess. Providing portable equipment and specifying areas for activities that dominate the elementary school playground during recess may be two approaches to increase recess physical activity levels, though further research is needed to evaluate the short and long-term impact of such strategies.

## Background

Physical activity engagement during childhood is important for growth, development and health. Habitual physical activity is associated with numerous health benefits in children, with observational studies suggesting greater health benefits are associated with higher levels of physical activity [1]. However, there is widespread concern that children are not engaging in sufficient activity to benefit health [2]. Efforts to increase physical activity in children are important for public health objectives, and schools are one particular setting that can be targeted for intervention implementation [3].

One context within schools that provides a valuable opportunity for the promotion of and engagement in physical activity is school recess (playtime in the United Kingdom); contributing up to 40% towards children's recommended daily physical activity [4]. In the United Kingdom, recess is mandatory and can account for up to 25% of the school day. Moreover, European studies have reported that children spend between 30-105 minutes in recess a day [5,6]. Recess is a daily free play opportunity where children can choose the play activities that they engage in. Interventions implemented during recess that involved modifying the environment have included games equipment [6,7] and playground markings [8,9]. Though a systematic review noted that the level of evidence for such interventions and their effect on physical activity levels are limited [3], some data suggest that increases may be sustained 6-months post-intervention [10]. However, with the observed effects differing between studies, it is likely that other factors may influence the intervention effects. Consequently, there is a need to establish what variables are associated with activity during recess [11]. Such information is likely to inform future intervention designs as the context in which the behaviour occurs is an important consideration.

Factors associated with physical activity are often termed "correlates" [12,13]. Reviews of the correlates of physical activity [12] and sedentary behaviour [14] have identified a range of multi-dimensional factors that can be targeted within interventions to increase activity or decrease sedentariness in children. Only two studies to date have investigated correlates specifically in a recess context. Cardon et al. [15] found that a hard playground surface was a significant predictor of preschool boys' physical activity, expressed as steps per minute, while for girls the presence of fewer supervisors was a positive predictor. Playground markings and toys, for example, were not significant predictors [15]. Haug et al. [16] assessed school facilities such as soccer fields, green spaces and sledding hills using self-report to assess physical activity. While children who attended a secondary school with a larger number of outdoor school facilities had higher odds of being physically active compared to children attending schools with fewer facilities, no significant associations were observed in primary school children. No data currently exist on correlates of sedentary activity during recess, and greater information concerning moderate or vigorous physical activity is needed to inform future activity promotion efforts.

Correlates of youth physical activity have been integrated in to a conceptual framework with the aim of guiding interventions and programmes [17]. The Youth Physical Activity Promotion Model (YPAPM) highlights how the demographics of the population affect the likelihood of engaging in physical activity (predisposing factors), the significant others that can reinforce physical activity behaviour (reinforcing factors), and characteristics of individuals and their environments which allow them to be active (enabling factors) across multiple levels of influence [17]. Emphasis is placed on the variables that are most amenable to change within the population of interest. As such, research pertaining to enabling and reinforcing factors that influence activity during recess is needed, as influencing these variables may impact on the activity of a large number of children due to the group setting in which the behaviours take place [18].

The aim of this study, therefore, was to investigate the association between a range of enabling and reinforcing recess variables and children's sedentary, moderate and vigorous physical activity in this context.

## Methods

### Participants

One hundred and fifty-two children (62 boys, 90 girls) aged 9-10 years from 8 elementary schools in one large city in North West England returned written informed parental consent and child assent to participate in the Active City of Liverpool, Active Schools and SportsLinx (A-CLASS) Project. Briefly, the A-CLASS Project is a longitudinal, multidisciplinary study of the effects of after-school clubs and a lifestyle intervention on children's recess and daily physical activity, fundamental movement skills (FMS), and laboratory-conducted markers of health. Full details concerning the recruitment of children in to the project have been described elsewhere [19]. For the purpose of this study, cross-sectional data collected during school time between June and July 2007 are reported. The research design and protocol received ethical approval from the Liverpool John Moores University Ethics Committee.

### Instruments

#### Direct Observation

Children's physical activity levels during recess were assessed using the System for Observing Children's Activity and Relationships during Play (SOCARP) [20]. SOCARP is a valid and reliable tool that enables researchers to simultaneously observe and record children's physical activity levels, social group sizes, activity types and social interactions during school recess. The validity of the physical activity variables have previously been demonstrated using heart rate monitoring [21] and uni-axial accelerometry [20]. The system uses time sampling techniques where a 10 second observe period is followed by a 10 second record period for each target child observed during recess. The target child is observed for 10 consecutive minutes (3 observations/min for 10 min of observations = 30 intervals) before the next child is located and observed. Target children are randomly selected children who are representative of the population under study. The children were observed once in a randomly selected recess period. At the end of each interval, data are recorded concerning children's physical activity levels, social group sizes, activity types and social interactions. In addition, SOCARP provides data on the availability of equipment and the number of adults supervising recess. These data are collected at the end of each target child's observation period (i.e. every 10 minutes during recess).

For the purpose of this study, SOCARP was used to generate the physical activity variables. The recorded physical activity code, namely lying down, sitting, standing, walking (moderate) and vigorous, reflect the activity level and/or body position of the child at the end of each interval [20]. If children were in transition between activity codes on the observe prompt, the code for the higher category was recorded. In situations where children were engaged in active behaviors whilst in sedentary positions (e.g. bouncing a ball on the spot whilst standing), they were coded as active [20]. In order to be classed as vigorously active, the child had to to expend more energy than they would for an ordinary walk. The percentage of time spent in the lying down, sitting, standing, moderate and vigorous activity codes was determined for each observed child and used in subsequent analyses.

#### Reliability

All data were collected by the primary author and one additional trained observer. Training consisted of developing familiarity with the systems protocol, memorizing observational categories and codes, using instrument notation and discriminating between variables within the different categories. Observers practiced coding videotaped examples of children's play behavior during recess, and feedback was provided on the results. The reliability assessment involved the observers coding videotapes simultaneously and independently of the primary author. Reliability criteria were set at 80% using interval-by-interval agreement for each category [22]. Observer reliability for the physical activity category was 89%. Training required 25 hours to reach acceptable inter observer criteria on the whole system (4 categories).

#### Anthropometry

Body mass was measured to the nearest 0.1 kg using a calibrated mechanical flat scale (Seca, Birmingham, UK). Stature and sitting height were measured to the nearest 0.1 cm using the Leicester Height Measure (Seca, Birmingham, UK). BMI was calculated using (mass (kg)/stature^2 ^(m)), and children were classified as normal weight or overweight using age-specific UK cut-points [23]. All measurements were taken by International Society for the Advancement of Kinanthropometry trained research staff using standard procedures.

#### Recess duration

This was defined as the time the school bell rang to start recess to the time it rang to conclude recess. Data were collected by the primary author who recorded these times on all days in all participating schools. All schools had a morning (mean = 16.4 ± 2.2 min) and lunch recess (mean = 60.6 ± 4.7 min), while 4 schools also had an afternoon recess period (mean = 13.7 ± 2.2 min). The time spent in all daily recess periods was added together to provide a total daily recess duration (mean = 83.8 ± 8.5 min).

#### Temperature

This was measured 5 minutes prior to a scheduled recess using a portable standard thermometer (GH Zeal Ltd., London, UK). For consistency across recess periods, the centre point of the play space available to the children in each school was determined and used for subsequent measurements. This point was not under cover or in the shade. Temperatures ranged from 12 to 27°C during data collection.

### Playground variables

#### Playground size

Google™ Earth Pro (GEP) software was used to provide an estimate of the playground spatial area (m^2^) at each of the schools in the project using aerial pictures of the playgrounds and the polygon measurement tool. Only one school had a grassy area within the school boundary, and children were not allowed on to this area on the days that observations took place; therefore this space was not measured or used in subsequent analyses. Playground sizes ranged from 1637.1 to 2392.9 m^2^.

#### Play space

The number of children enrolled in each school was provided by schools during researcher visits. Average play space per child was calculated by dividing the number of children in the school by the playground size available for use during recess.

#### Physical playground features

The number of fixed equipment features (e.g. soccer posts, wooden features, climbing frames), playground markings, and seating (e.g. individual seats, covered seating areas) were counted and recorded during each school visit. The number of features in each of these categories was summed, and the three values (fixed equipment, markings and seating) were used in subsequent analyses.

### Procedure

Each school was visited on three consecutive school days during the data collection (24 days total). Data were collected from all daily school recess periods. Four schools had two daily recess periods (morning, lunch), while four schools had 3 daily recess periods (morning, lunch, afternoon). Observers arrived at schools prior to morning recess and obtained information about the playground environment. On the first day at each school, data concerning precise recess times were also obtained. Five minutes prior to the start of recess, observers collected temperature data then positioned themselves in an inconspicuous place, such as the perimeter of the playground, where they could view the entire playground. Observations commenced once a previously selected child entered the playground. This child was observed for 10 minutes; after which, the focus moved on to the next selected child. Observers concurrently observed different children. This process continued until the end of recess. Data were collected "live" on 12 days as 4 schools did not permit video recordings to be made, and 12 days were video recorded using a DV tape camcorder for analysis later in the university laboratory. Children were accustomed to being videoed due to the assessment of FMS, which was part of the larger project.

### Data Analysis

Full data were obtained for 128 children (50 boys, 78 girls) and used in subsequent analyses. Reasons for missing data included absence from school on testing days and children not entering the playground area due to disciplinary reasons. Descriptive statistics were calculated to describe the final sample. Initial exploratory analyses were conducted to establish whether significant differences occurred between the physical activity variables and the recess period in which the child was observed. These data were analysed using SPSS v17, and the significance level set at *p *≤ 0.05.

To account for the nested nature of the data and the hierarchical structure of the YPAPM [17], multilevel models were used to analyse the data. A two-level structure was used, with children designated as the first level unit and schools designated the second level unit. This design captures individual variation in the data, and the effect of the context in which the behaviour occurs [24]. Data were analyzed using MLwiN 1.10 software (Institute of Education, University of London, UK).

A prediction model was used to identify which variables were significantly associated with the outcome variables. The outcome variables were the proportion of time spent in sedentary (sum of lying, sitting and standing codes [21]), moderate and vigorous physical activity during recess. Level 1 variables entered in to the model were BMI, age (both continuous variables) and sex (dichotomous variable). Level 2 variables entered were temperature, recess duration, play space, playground physical features, supervision (all continuous variables) and equipment provision (dichotomous variable). A backwards selection procedure was used for the analysis, where the variable least strongly associated with the outcome variable was removed [24]. Variables were retained in the model if they significantly predicted the outcome variables, and remained significant when additional variables were retained in the models. Three models were constructed in total. Regression coefficients were assessed for significance using the Wald statistic [24]. Significantly associated variables were retained in the final model, with statistical significance set at p ≤ 0.05 [24].

## Results

Table 1 reports the descriptive data for the whole sample. Exploratory analyses of variance revealed no significant differences in sedentary, moderate and vigorous activity across the recess periods (*p *> 0.05). Information concerning the predictor variables entered in to the analyses is shown in Table 2.

**Table 1:** Descriptive data for the sample (mean (SD)) unless stated

	All children (n = 128)
**Age (yr)**	10.4 (0.3)
**Body mass (kg)**	39.6 (9.3)
**Stature (cm)**	142.4 (7.2)
**Body mass index (m.kg^-2^)**	19.3 (3.3)
**Overweight (%)**	30.7
**Obese (%)**	10.2
**% Sedentary during recess**	42.7
**% Moderate during recess**	39.4
**% Vigorous during recess**	17.8

**Table 2:** Information concerning predictor variables used in the analyses

Variable	Additional Information	All (n = 128)	
		Range	Mean (SD)
**Recess duration (min)**	Total recess time per day	75-105	83.8 (8.5)
**Equipment^1^**	Portable equipment provided either by the school or the children for use during recess. Includes bats, balls, jump rope.	Yes/No	N/A
**Temperature (°C)**	Temperature recorded at the start of the recess period	12-27	19 (4.0)
**Playground size (m^2^)**	Size of available play space during recess	1637.1-2392.9	2017.5 (229)
**Play space**	Average play space per child based on number on roll and playground size	4.6-13.8	6.2 (2.7)
**Number on roll**	Total number of children attending the school	133-435	359.8 (89.8)
**Fixed equipment**	Total number of man-made physical structures in the playground available for use during recess (e.g. soccer goals, basketball nets)	1-6	2.8 (1.7)
**Playground markings**	Total number of playground markings painted on the playground surface	1-8	4.4 (2.2)
**Seating**	Number of fixed benches on the playground for children to use during recess	3-11	4.8 (2.3)
**Supervision**	Number of adult supervisors on the playground recorded at the end of each scan	1-7	3.2 (1.7)

Abbreviations: N/A not applicable.

^1 ^Categorical variable

Results from initial analyses showed statistically significant differences in physical activity levels between schools prior to predictor variables being added to the models for each intensity. The total variance explained by differences between schools was 11.9% for sedentary activity, 9.0% for moderate activity and 20.7% for vigorous activity. The results of the multilevel models for sedentary behaviour, moderate, and vigorous activity during recess are shown in Tables 3 and 4, respectively. The significant variables accounted for 17.6%, 7.7% and 24.2% of the variance in sedentary, moderate and vigorous activity, respectively [23].

**Table 3:** Factors influencing sedentary activity during recess

	Model 1	Model 2		
**Correlate**	**β (SE)**	**β (SE)**	**95% CI**	***p *value**

Constant	42.15 (3.07)	57.42 (5.66)	46.33 to 68.51	< 0.001
Sex^1^	NE	13.82 (3.41)	7.14 to 20.5	< 0.001
Play space	NE	-2.70 (0.60)	-3.88 to -1.52	< 0.001
Equipment^2^	NE	-8.15 (4.15)	-16.28 to -0.02	0.049
				
% Total variance explained	N/A	17.6		
				
**Random**				
School Level	50.5 (37.76)	21.34 (16.35)		
Child Level	372.87 (48.95)	327.38 (41.58)		
Total variance	423.37	348.72		
Deviance	1095.08	1069.99		
ICC	0.119	0.061		

^1 ^Reference category for sex is boys.

^2 ^Reference category is no equipment

Abbreviations: N/A - not applicable; NE - not entered in to model; β = Regression coefficient; SE = Standard Error; CI = Confidence Interval

**Table 4:** Factors influencing moderate and vigorous activity during recess

	Moderate					Vigorous			
	
	Model 1	Model 2				Model 1	Model 2		
	
Correlate	β (SE)	β (SE)	95% CI	*p *value	Correlate	β (SE)	β (SE)	95% CI	*p *value
Constant	39.27 (1.41)	33.67 (3.29)	27.22 to 40.12	< 0.001	Constant	18.38 (2.49)	22.79 (6.81)	9.44 to 36.14	< 0.001
Equipment^2^	NE	6.91 (3.42)	0.21 to 13.61	< 0.05	Sex^1^	NE	-8.22 (2.18)	-12.49 to -3.95	< 0.001
					Temperature	NE	-0.65 (0.28)	-1.20 to -0.10	0.02
					Play space	NE	2.02 (0.42)	1.20 to 2.84	< 0.001
									
% Total variance explained	N/A	7.7			% Total variance explained	N/A	24.2		
									
**Random**					**Random**				
School Level	24.38 (15.13)	20.29 (14.33)			School Level	39.51 (24.86)	14.39 (11.26)		
Child Level	245.52 (31.81)	228.78 (30.33)			Child Level	151.29 (19.86)	130.26 (16.54)		
Total variance	269.9	249.07			Total variance	190.8	144.65		
Deviance	1034.32	1028.86			Deviance	987.09	955.72		
ICC	0.090	0.081			ICC	0.207	0.099		

^1 ^Reference category for sex is boys.

^2 ^Reference category is no equipment

Abbreviations: N/A - not applicable; NE - not entered in to model; β = Regression coefficient; SE = Standard Error; CI = Confidence Interval

Sex was a significant positive predictor of sedentary activity and a negative predictor of vigorous physical activity. Girls engaged in 13.8% more sedentary activity and 8.2% less vigorous activity than boys during recess. Equipment provision was a significant negative predictor of sedentary activity and positive predictor of moderate activity. Children with no equipment provision during recess engaged in 8.2% more sedentary activity and 6.9% less moderate activity than children who were provided with equipment during recess. Play space was a significant negative predictor of sedentary activity and a positive predictor of vigorous activity. As play space per child increased, sedentary activity decreased and vigorous activity increased. Temperature was also a significant negative predictor of vigorous activity.

## Discussion

The aim of this study was to investigate the association between a range of enabling and reinforcing recess variables and children's sedentary, moderate and vigorous physical activity in this context. The results revealed that girls engaged in 13.8% more sedentary activity and 8.2% less vigorous activity than boys during recess. This supports previous research highlighting how boys are more physically active than girls when measured using objective [8,10,25] and self-report measures [16].

Play space was significantly negatively associated with sedentary activity and positively associated with vigorous activity. Previous studies have noted that more space available per child is associated with recess physical activity [15] and that children are more active in spacious compared to restricted environments [26]. Conversely, Sallis et al. [27] found that area size was not significantly associated with physical activity, though their study assessed available space in different school ground areas rather than the space available per child. The current study's findings may partly be explained by the types of recess activities the children engaged in. Armitage [28] stated that soccer often dominates over half the elementary school playground yet it is played by approximately one quarter of the school population, leading to the remaining children situating themselves around the playground perimeter and engaging in inactive behaviours. As such, recess strategies to increase physical activity could consider reducing the dominance of soccer by allocating specific areas for this activity [7,10], which could enable more children to be active as they have more space on the playground.

Interventions conducted in recess have used games equipment to enable greater engagement in active behaviours, with both positive effects [7,10,29] and no effects [30] found. This present study found that equipment availability was a positive predictor of moderate and negative predictor of sedentary activity. Interestingly, this contrasts Haug et al. [16] and Zask et al. [11] who found that, in elementary school children, equipment availability was not significantly associated with moderate or vigorous physical activity. Zask et al. [11] did find, however, that the ball-to-child ratio was a significant predictor of vigorous physical activity, whilst Willenberg et al. [31] found that more children engaged in vigorous physical activity when loose equipment (e.g. bats, balls, jump ropes) was provided. It may be that equipment enables children to be active and certain equipment simulates greater activity compared to others. For example, soccer balls may stimulate continuous physical activity engagement, while jump rope could facilitate intermittent bouts of activity. Overall, these findings suggest that the use of equipment may be a simple way to enable children to be active during recess, though further research is needed to determine how the availability of equipment, the type of equipment available and the amount of equipment provided influence physical activity and sedentary behaviours. Such information could inform future recess interventions.

In the present study, temperature was negatively associated with vigorous activity during recess. This supports Ridgers et al. [20] and Farley et al. [32], who collected data in June-July in North-West England and over a 2-year period in New Orleans, USA, respectively. However, these findings contrast Zask et al. [11], who collected data in February-April in Australia. A recent review by Carson and Spence [33] noted that meteorological variables have not generally been specifically researched in physical activity studies, though seasonal variations suggest that higher activity levels occur during summer months. Duncan et al. [34] found that boy's activity increased as temperature increased, whilst in adolescents increasing temperatures are associated with more physical activity sessions [35]. Notably, these latter studies have focused on habitual physical activity, where children choose to be outside and are likely influenced by weather conditions. This differs from recess in the UK where children are required to be on the playground during recess with the exception of when they eat their lunch or when it is raining. It is possible that whilst warmer weathers encourage children to spend time outdoors, in a context where children already access the outdoor environment temperature may be an important consideration when assessing physical activity levels and implementing interventions. As such, it is recommended that recess studies should report temperature and weather to aid cross-study comparisons.

Interestingly, physical playground features were not associated with physical activity at any intensity. This supports the findings of Cardon et al. [15] but contrasts previous research that have changed the physical environment to enable physical activity engagement [9,10]. It is possible that the measure used in this study was not sensitive enough to detect associations with physical activity. Alternatively, it may be that the age of the playground markings and features that are associated with physical activity during recess, as recent data have suggested that physical playground features have a positive effect on children's activity, but the effects begin to decrease after 6 months [36]. More research in this area is needed.

The number of supervisors on the playground was also not associated with physical activity during recess. Mixed findings have been reported concerning this issue to date [11,20,27,31]. In the present study, supervising adults generally allowed the children to engage in free play, and were not involved in the organisation of children's games. The presence of adults could be one potential strategy to decrease children's sedentary activity and reinforce physical activity [17], though since recess is an opportunity for activity engagement which is largely free from adult control, more research is needed to identify factors in this context which could enable and reinforce children's activity levels.

There are several limitations that warrant attention. Firstly, the sample size at the school level is relatively small, though multilevel modelling analyses have been used previously on a similar number of schools [19]. In addition, the playgrounds were not broken down in to specific areas (e.g. fixed equipment, soccer pitches), which has been the approach adopted in European and American studies and effects comparability across studies. In this study, children only had access to one playground which contained different fixed structures, which made it difficult to break the available outdoor space in to specific areas. Thirdly, collected data during short recess periods may not be representative of activity levels and behaviour during longer recess periods, though research suggests that physical activity data collected within discrete periods of the day may be more consistent in the school environment [37]. Notably, no differences were found between the physical activity variables and the recess periods in this study. Lastly, it should also be noted that the three outcome variables of interest are correlated, therefore engagement in one physical activity intensity will influence the proportion of time that children are active at another intensity.

## Conclusions

Understanding variables that influence children's physical activity levels within the school environment may help to inform future interventions. Focusing specifically on recess, which is a free play opportunity during the school day, this study found that equipment, play space per child and temperature influenced physical activity levels. Providing portable equipment and specifying areas for activities that dominate the elementary school playground during recess may be two approaches to increase recess physical activity levels. Weather conditions may also need to be accounted for when assessing recess activity levels to identify periods of lower activity during the school year and determining whether interventions were successful in increasing physical activity and decreasing sedentary activity. More research concerning predisposing factors [17] and their associations with recess physical activity is needed.

## Competing interests

The authors declare that they have no competing interests.

## Authors' contributions

GS conceived the study and secured the funding. NDR and GS contributed to the planning and design of the study. NDR collected the data, and conducted the data manipulation. NDR and SJF conducted the analyses. NDR wrote the manuscript and SJF and GS supplied comments on the manuscript. All authors read and approved the final version of the manuscript.
